# Gα-cAMP/PKA pathway positively regulates pigmentation, chaetoglobosin A biosynthesis and sexual development in *Chaetomium globosum*

**DOI:** 10.1371/journal.pone.0195553

**Published:** 2018-04-13

**Authors:** Yang Hu, Xiaoran Hao, Longfei Chen, Oren Akhberdi, Xi Yu, Yanjie Liu, Xudong Zhu

**Affiliations:** 1 Department of Pathogen Biology, School of Basic Medical Sciences, Tianjin Medical University, Tianjin, China; 2 National Experimental Teaching Demonstrating Center, School of Life Sciences, Beijing Normal University, Beijing, China; 3 Department of Microbiology, College of Life Sciences, Nankai University, Tianjin, China; 4 Beijing Key Laboratory of Genetic Engineering Drug and Biotechnology, Institute of Biochemistry and Biotechnology, School of Life Sciences, Beijing Normal University, Beijing, China; University of California Riverside, UNITED STATES

## Abstract

Sensing the environmental signals, the canonical Gα-cAMP/PKA pathway modulates mycelial growth and development, and negatively regulates some secondary metabolism in filamentous fungi, *e*.*g*. aflatoxin in *Aspergillus nidulans*. Here we report the characterization of this signaling pathway in *Chaetomium globosum*, a widely spread fungus known for synthesizing abundant secondary metabolites, *e*.*g*. chaetoglobosin A (ChA). RNAi-mediated knockdown of a putative Gα-encoding gene *gna-1*, led to plural changes in phenotype, *e*.*g*. albino mycelium, significant restriction on perithecium development and decreased production of ChA. RNA-seq profiling and qRT-PCR verified significantly fall in expression of corresponding genes, *e*.*g*. *pks-1* and *CgcheA*. These defects could be restored by simultaneous knock-down of the *pkaR* gene encoding a regulatory subunit of cAMP-dependent protein kinase A (PKA), suggesting that *pkaR* had a negative effect on the above mentioned traits. Confirmatively, the intracellular level of cAMP in wild-type strain was about 3.4-fold to that in *gna-1* silenced mutant pG14, and addition of a cAMP analog, 8-Br-cAMP, restored the same defects, *e*.*g*., the expression of *CgcheA*. Furthermore, the intracellular cAMP in *gna-1* and *pkaR* double silenced mutant was approaching the normal level. The following activity inhibition experiment proved that the expression of *CgcheA* was indeed regulated by PKA. Down-regulation of *LaeA/VeA/SptJ* expression in *gna-1* mutant was also observed, implying that Gα signaling may crosstalk to other regulatory pathways. Taken together, this study proposes that the heterotrimeric Gα protein-cAMP/PKA signaling pathway positively mediates the sexual development, melanin biosynthesis, and secondary metabolism in *C*. *globosum*.

## Introduction

*Chaetomium* fungi are commonly found in the environment with more than 350 species described in this genus [1]. Relying on robust enzyme activities, this group of fungi are able to thrive on a broad range of substrates, *e*.*g*. lignocellulose, and are widely distributed in soils, marine environments, animal dung, hair, textiles, plant seeds and some other substrates rich in cellulose [2, 3]. Some species of the genus *Chaetomium* are reported to be able to cause plant diseases and human infections and can also be plant endophytes [4]. Recently the genus *Chaetomium* has received attention for its capacity to produce a myriad of secondary metabolites that supposedly favor thriving in varied ecological niche [5]. More than 200 small secondary metabolite compounds have been reported from *Chaetomium* spp., among which chaetoglobosins are well known for their robust cytotoxic bioactivity and potential pharmaceutical significance [1, 6].

Chaetoglobosins are grouped into the cytochalasin family of natural products and are actually polyketide derivatives found in fungi [7]. They have unique biochemical property of binding eukaryotic actin proteins, disturbing the normal actin network in the cell. Thus, they display strong cytotoxicity against tumor cell lines [8, 9], phytotoxicity on numerous plants [10], immunomodulatory activities [11], and antifungal [12] and nematicidal activities [13]. This broad range of cytotoxicity has significance for drug development. To date, more than eighty chaetoglobosins have been reported from different genera of filamentous fungi [9, 10, 12], including species of the genus *Chaetomium*. Structurally, chaetoglobosins share a macrocyclic polyketide core associated with an isoindolone moiety. In *Penicillium expansum*, the core structure is synthesized by a hybrid megasynthetase, *i*.*e*. a polyketide-nonribosomal peptide synthase (PKS-NRPS) named CheA, and a stand-alone enoyl reductase named CheB [14]. The genes are located within a single gene cluster. The macrocyclization step is finished via the Diels-Alder reaction. Nonetheless, we previously observed in *C*. *globosum* NK102 that a polyketide synthase gene, *pks-1*, was required for both colonial pigmentation and biosynthesis of chaetoglobosin A (ChA) via an unknown mechanism [15].

The capability of an organism to respond to external stimuli using signal transduction is critical for concerting metabolism and development [16]. In eukaryotic organisms, the canonical heterotrimeric G proteins, composed of α, β, and γ subunits, are a major player in regulating a variety of cellular processes through a signaling cascade to intracellular effectors, such as adenylate cyclase, phospholipases, and ion channels. In filamentous fungi, sensing a depletion of carbon source or amino acids by a G-protein coupled receptor (GPCR) will activate the coupled Gα subunit of the G protein complex that in turn transfers the signal to adenylyl cyclase, to regulate the *in vivo* cyclic adenosine monophosphate (cAMP) levels. Intracellular cAMP binds to the regulatory subunits of cAMP-dependent protein kinase A (PKA), and then phosphorylates various protein targets in the pathways of fungal growth, development and the biosynthesis of some secondary metabolites [17–21]. For instance, the pathway negatively regulates the synthesis of aflatoxin in *Aspergillus nidulans*. However, the role of G protein-cAMP/PKA signaling pathway in the biosynthesis of ChA has not been defined in *C*. *globosum*.

As part of our ongoing effort on the regulation of secondary metabolism in *C*. *globosum*, we started with the definition of the role of G protein-cAMP/PKA pathway in the biosynthesis of ChA. The fungal strain *C*. *globosum* NK102 was formerly isolated as a high-yield ChA producer which could use cellulose as a sole carbon source [15, 22, 23]. In our laboratory, ChA had exhibited strong nematicidal activity against *Meloidogyne incognita* (the lethal concentration 50 (LC_50_) is 77.0 μg/mL) [13] and a severe inhibitory activity against the plant pathogenic oomycete *Pythium ultimum* (unpublished data). To gain insight into the regulation mechanism of ChA biosynthesis in *C*. *globosum*, we knocked down the gene *gna-1* (CHGG_03321), putatively encoding a group I Gα protein, and the gene *pkaR* (CHGG_00688), encoding the regulatory subunit of the cAMP-dependent PKA. Using this established RNA interference (RNAi) strategy [15, 24], we demonstrated that the canonical G protein-cAMP/PKA pathway plays a pivotal role in the production of ChA in *C*. *globosum* NK102. The effects of *gna-1* and *pkaR* are presented below.

## Materials and methods

### Fungal strains and growth conditions

The wild-type strain *C*. *globosum* NK102, isolated and stock by our laboratory was used as the host strain for RNAi experiments [15]. NK102 was grown on potato dextrose agar (PDA) at 28°C. For making the protoplasts of NK102, potato dextrose broth (PDB) was inoculated with 10^6^ spores and incubated for 3 days in a rotary shaker at 28°C and 180 rpm. For DNA or RNA isolation, 5-mm agar plaques containing the fungal hypha inoculated in PDB were incubated for 5 days in a rotary shaker at 28°C and 180 rpm. For RNA-seq analysis, 5-mm agar plaques with hypha of the wild-type or transformants were inoculated in MCC medium (per liter: 10 g microcrystalline cellulose, 1.4 g (NH_4_)_2_SO_4_, 2.0 g KH_2_PO_4_, 0.3 g urea, 0.3 g MgSO_4_·7H_2_O, 0.3 g CaCl_2_, 1.0 g peptone, and trace elements, *i*.*e*., 5 mg FeSO_4_·7H_2_O, 1.56 mg MnSO_4_·H_2_O, 1.67 mg ZnCl_2_, 2.0 mg CoCl_2_) [2] and incubated for 9 days, shaking at 28°C, 180 rpm. For high-performance liquid chromatography (HPLC) analysis, strains were cultured in 200 ml PDB for 8 days with shaking at 28°C, 180 rpm. For cAMP assays for wild-type and mutant pG14 and pGP6, strains were cultured in 200 ml MCC for 72 hours with shaking at 28°C, 180 rpm.

### Plasmid construction for RNA interference

**Fig 1:**
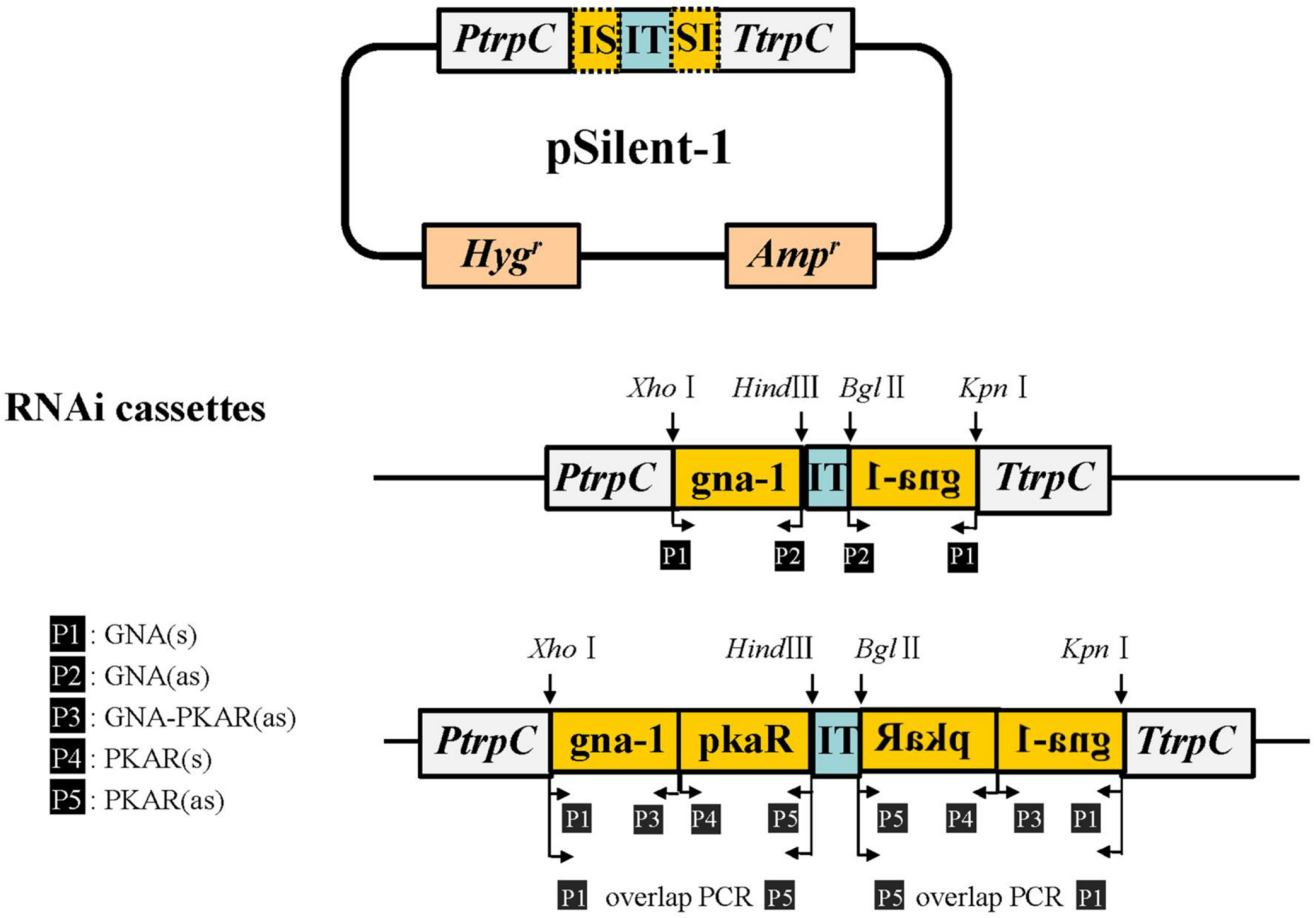
Construction of RNA interference cassettes. RNA interference cassettes were constructed based on the pSilent-1 plasmid [25]. The inserted fragments were obtained by PCR following restriction enzyme digestion. Primers are indicated in black boxes. IS, inserted fragment; IT, intron 2 of cutinase (CUT) gene from *Magnaporthe oryzae*; PtrpC, promotor of *trpC* from *A*. *nidulans*; TtrpC, *trpC* terminator of *A*. *nidulans*; *Hyg*^*r*^, hygromycin resistance; *Amp*^*r*^, ampicillin resistance. Restriction enzyme sites are also indicated.

An RNAi cassette was constructed based on the pSilent-1 vector, a kind gift from Dr. Jinzhu Song (Harbin Institute of Technology, China), which has been used in a variety of fungi [25]. Plasmids for RNA interference were constructed as described previously [15]. Briefly, the *gna-1* knock-down plasmid was produced by inserting a 284-bp fragment located at the 5′ end of *gna-1* into the *Xho* I-*Hind* III restriction enzyme sites of pSilent-1, and a reverse sequence into the *Bgl* II-*Kpn* I restriction enzyme sites of pSilent-1. Both inserted fragments were amplified from NK102 genomic DNA using primers GNA1(s)/GNA1(as). The resulting recombinant was designated pGNA-1 (Fig 1). The same strategy was used to create plasmid pGNA-PKAR for simultaneous knock-down of the expression of *gna-1* and *pkaR*. The 210-bp fragment of *pkaR* and the 320-bp fragment of *gna-1* were amplified using primers PKAR(s)/PKAR(as) and GNA1(s)/GNA-PKAR(as), respectively. The *gna-1*-*pkaR* fusion fragment was obtained by overlap PCR using the primers GNA1(s)/PKAR (as) (Fig 1). All primers are detailed in S1 Appendix.

### Fungal transformation and transformants screening

Protoplast preparation and transformation were performed as previously described [15]. Briefly, fresh mycelia were collected by centrifugation, washed three times with sterile distilled water, and rinsed with 0.7 M NaCl. The cells were incubated with 20–30 ml of 0.7 M NaCl containing 10 mg/ml snailase and 2 mg/ml cellulase (Solarbio, Beijing, China) at 35°C for 2 h. The released protoplasts were filtered and subsequently centrifuged at 2000 *g* for 10 min at 4°C. The collected protoplasts were washed with STC (1.2 M sorbitol, 10 mM CaCl_2_ and 10 mM Tris-HCl, pH 7.5) and adjusted to a concentration of 10^7^−10^8^ protoplasts per milliliter. A 100-μl suspension of *C*. *globosum* protoplasts was gently mixed with 20–30 μl (0.4–1.0 μg/μl) circular plasmid DNA. The mixture was kept on ice for 20 min before addition of 60% PEG3350 (60% polyethylene glycol 3350, 50 mM CaCl_2_ and 10 mM Tris-HCl, pH 7.5) and completion of the usual transformation and plating protocols [26]. After incubation for 12–16 h at 28°C, plates (each containing 10 ml regeneration medium) were overlaid with 10 ml of 1% agar containing hygromycin B at 200 µg/ml and incubated at 28°C. Transformants appeared 3–7 days after plating.

Transformants were selected on PDA plates containing 100 μg/ml hygromycin B. For screening of the correct transformants, the hygromycin B-resistant cassette, consisting of the hygromycin B phosphotransferase gene *hph*, was amplified by a diagnostic PCR using primers hyg(s)/hyg(as) (S1 Appendix). For further confirmation, genomic DNA was extracted and subjected to Southern blot analysis as previously described [15]. A 284-bp *gna-1* fragment and the linearized pSilent-1 vector digested by *Xho* I were separately labeled as probes. Experiments involving DNA labeling, hybridization and detection were carried out according to the instructions of the DIG High Prime DNA Labeling and Detection Starter Kit II (Roche China, Shanghai, China).

### RNA isolation and quantitative real-time (qRT)-PCR

Total RNA was extracted from the lyophilized and ground mycelium using a TRIzol kit (Invitrogen, CA, USA), and was then treated with RNase-free DNase (Takara Inc, Dalian, China) to remove possible contaminant DNA. The first-strand cDNA was generated by reverse transcription in a 20 μl reaction using Moloney Murine Leukemia Virus (M-MLV) RTase cDNA synthesis kit (Takara Inc.). Quantitative real-time PCR was performed by Mastercycler PCR (Eppendorf, Hamburg, Germany). Each reaction of 20 μl PCR was performed with SYBR Green I PCR master mix (Roche China, Shanghai, China). Reactions were set up in three replicates per sample. Controls without addition of the templates were included for each primer set. PCR cycling parameters were: pre-incubation at 94°C for 10 min, followed by 40 cycles of denaturation at 94°C for 15 s, annealing at 59°C for 30 s and extension at 72°C for 32 s. The qRT-PCR data were analyzed using the 2^-△△Ct^ relative quantification method [27] in the instrument’s software. The housekeeping gene encoding actin served as reference. The amplification efficiencies of the target and reference genes were compared at different template concentrations. The gene-specific pairs of primers used in the amplifications were: qGNA1(s)/qGNA1(as) for *gna-1*, qPKA(s)/qPKA(as) for *pkaR*, and qActin(s)/qActin(as) for the actin gene (S1 Appendix).

### Detection of chaetoglobosin A by HPLC

Each sample of liquid culture was extracted with an equal volume of chloroform: methanol (10:1, v/v). The organic phase was then transferred to a vacuum evaporator and concentrated under reduced pressure at 55°C until pellet formation. The residue was dissolved in 2 ml methanol, suspended and centrifuged at 12000 rpm for 10 min. The supernatant was filtered through a 0.45-μm Millipore filter and subjected to HPLC analysis on an Agilent 1100 HPLC system (Agilent Technologies, CA, USA) with a Kromasil C18 ODS column (4.6×250 mm, AKZO Nobel, Gland, Switzerland). The UV detection wavelength was set at 227 nm, and the sample flow rate was set at 1 ml/min. Standard ChA (Sigma, St. Louis, USA) served as control. For quantification of ChA, a standard curve was created with known concentrations of the standard sample.

### cAMP competitive enzyme immunoassay

To observe the effect of decreased activity of PKA on the cAMP, the *in vivo* concentration of cAMP from the wild-type, mutant pG14 and pGP6 were extracted and quantified as following: fresh mycelia were collected and frozen in liquid nitrogen. To extract cAMP, the frozen mycelia were grounded to a fine powder, weighed, suspended and lysed directly in 0.1M HCl (1:10, w/v) for 20 min. Details of intracellular cAMP extraction was followed as previously described[28]. The supernatant was recovered after centrifugation at 6000 ×g for 10 min. The cAMP concentration of the supernatant was measured by using the Direct cAMP enzyme immunoassay (EIA) kit CA-200 (Sigma-Aldrich, USA) according to the protocol supplied by the manufacturer.

### Effects of 8-Br-cAMP and H-89 on chaetoglobosin A biosynthesis

To observe the effect of the *in vivo* concentration of cAMP on the biosynthesis of ChA, PKA activator 8-Br-cAMP (8-bromoadenosine-3', 5’-cyclic monophosphate) (Sigma–Aldrich) was supplemented in 200 ml PDB medium at concentrations of 0, 1, 2, 5 and 10 mM. The effect of PKA inhibitor H-89 (N-[2-(p- Bromocinnamylamino) ethyl]-5-isoquinolinesulfonamide·2HCl hydrate) (Sigma–Aldrich) was also studied in the same method but at much lower concentrations (0, 1, 2, 5, 10 μM). Production of ChA biosynthesis was monitored on the relative expression of the gene CHGG_01239 (*CgcheA*) by qRT-PCR. The yield of ChA from the wild-type was quantified by HPLC.

### RNA-seq, data mining and gene ontology analysis

RNA-seq profiling was carried out by a commercial provider to monitor the consequences of the knock-down mutants of G protein. Illumina HiSeq™ sequencing of total mRNA from the wild-type or the transformant pG14 was conducted by BGI (Shenzhen, China; http://en.genomics.cn/navigation/index.action). The genome sequence of *C*. *globosum* NK102 was used as the reference for the analysis (unpublished data). P-values were used to evaluate expression differences at a statistically significant level [29]. A false discovery rate (FDR)-corrected P value <0.001 and an absolute log_2_Ratio value ≥1 were used to identify the differentially expressed genes (DEGs) and differentially expression tags (DETs).

## Results

### RNAi-mediated knockdown of *gna-1* and *pkaR* in *C*. *globosum* NK102

To investigate whether the secondary metabolite ChA of *C*. *globosum* was under the control of the G protein-cAMP/PKA signaling pathway, genes *gna-1* and *pkaR*, two key components in this pathway, were knocked down by RNAi. A single-copy homolog of *gna-1* (homolog of CHGG_03321 of *C*. *globosum* CBS 148.51), was found in the genome of *C*. *globosum* NK102. The 1265-bp gene *gna-1* was cloned from *C*. *globosum* NK102, subjected to sequencing and submitted to the GenBank database under accession number KC351752. A homolog of the gene *pkaR* (equivalent of CHGG_00688 of *C*. *globosum* CBS 148.51) was also defined as a single copy in the genome of *C*. *globosum* NK102 (GenBank accession no. KY990712). To obtain the knock-down mutant of gene *gna-1*, we transformed the protoplasts of *C*. *globosum* NK102 with the RNAi cassette pGNA-1 that contained two inverted 284-bp complementary fragments of the 5′ end of *gna-1* (Fig 1). Thirty purified hygromycin B-resistant transformants were obtained by PCR screening (See Experimental Procedures). Those transformants that underwent a genome integration event would produce a PCR fragment of the desired size, and were designated as pGs. Similarly, the transformants for knock-down of both *gna-1* and *pkaR* were generated using the RNAi vector pGNA-PKAR. Twenty-six hygromycin-resistant transformants were selected by PCR screening and designated as pGPs.

Southern blotting was performed as a further verification of the transformants (S2 Appendix). Genomic DNA of randomly picked transformants, pG3, pG14, pG17, pG18, pG21, and pG23 was prepared and digested by *Xba* I. There were two *Xba* I sites located in the interference cassette (2.7 kb) carried on pSilent-1, but not in the sequence of *gna-1* (S2 Appendix), thus an intact *gna-1* band and a 2.7 kb RNAi cassette insertion band should be detected in the mutants when probed with a 284-bp *gna-1* fragment (highlighted in green). All transformants were proved to be positive, ascertaining that the native copy of *gna-1* remained intact in the transformants. Similarly, Southern blotting of seven randomly selected transformants pGP1, pGP2, pGP3, pGP4, pGP5, pGP6 and pGP7, detected a 3.3-kb band that carried the entire *gna-1/pkaR* interference cassettes (S2 Appendix) in all the transformants. Among the transformants, pGP6 and pGP7 were confirmed to have a desired single integration of the vector. In previous work, we generated a control mutant Ct by transforming pSilent-1 alone in *C*. *globosum* NK102, and found that the vector itself had no discernible effect on the phenotype of the fungus [15].

To determine the copy number of the interference cassettes inserted in the pG transformants, genomic DNA was digested by *Xho* I, which has a single recognition site located in pGNA-1. Thus, two bands were expected for a single insertion event when the labeled pSilent-1 alone served as probe. As shown in Figure C in S2 Appendix, two bands were seen in the lanes of transformants pG14, suggesting a single copy of the interference cassette was inserted into the genome of these transformants, whereas more than two bands appeared in the lanes of pG3, pG17, and pG18, indicating multiple copies of interference cassettes had inserted in these transformants. No hybridization signal was detected in the lane of the wild-type strain.

### Decreased transcription of *gna-1* and *pkaR* in the knock-down mutants

**Fig 2:**
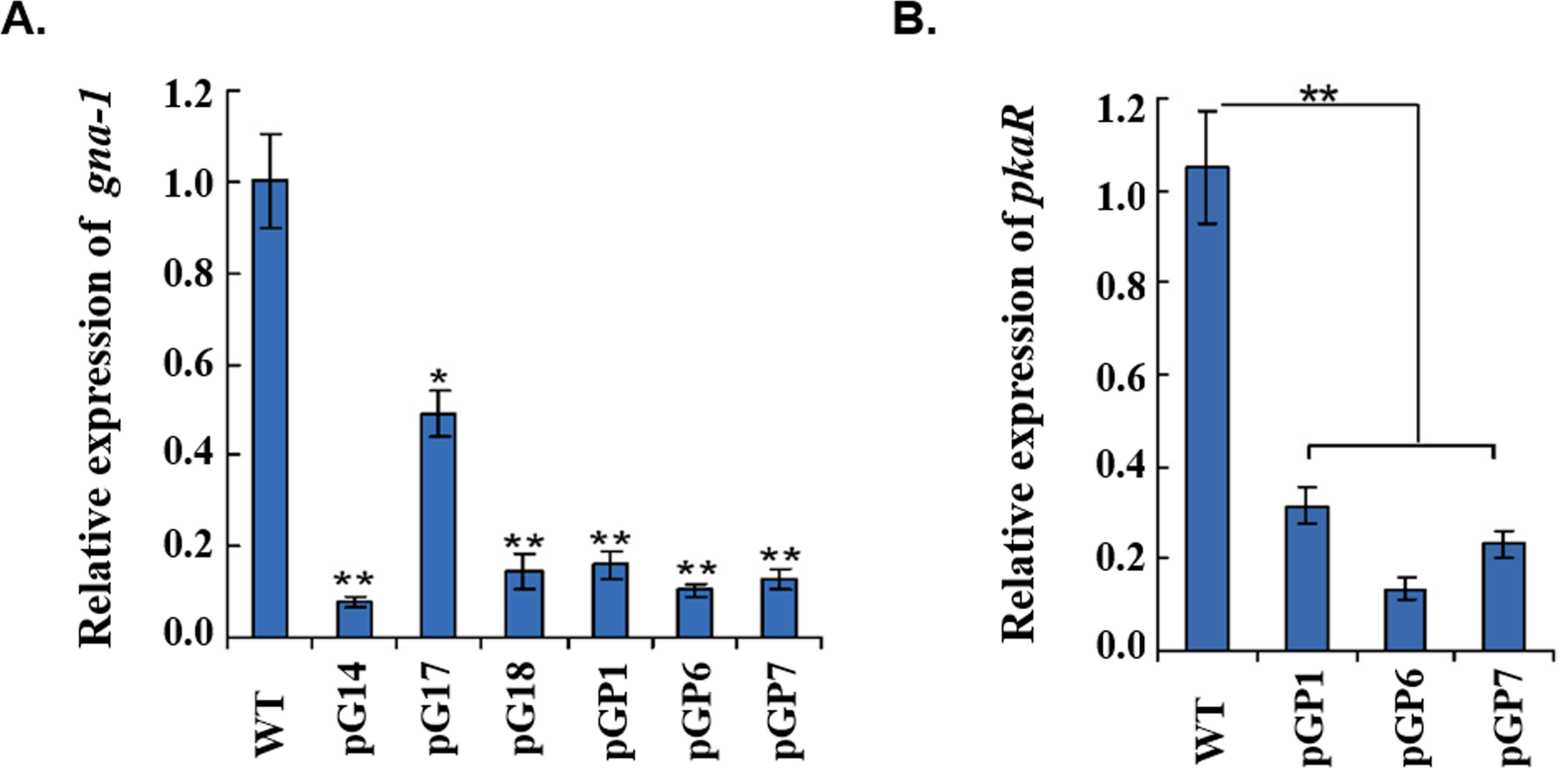
Gene expression of *gna-1* and *pkaR* in RNAi mutants. **(A**) Transcript levels of the *gna-1* were detected by qRT-PCR in the wild-type, pG and pGP transformants using the primers qGNA(s) and qGNA(as). **(B**) Transcript levels of *pkaR* were measured by qRT-PCR in the wild-type and pGP transformants using the primers qPKAR(s) and qPKAR(as). Transcripts of *gna-1* and *pkaR* were normalized against actin amplified with primers qActin(s) and qActin (as) (S1 Appendix). There is significantly difference between the mutant and wild-type as indicated by an asterisk (p-value <0.05 with T-test analysis) or by two asterisks (p-value <0.01 with T-test analysis). Experiments were performed in triplicate.

Expression of *gna-1* was examined by qRT-PCR in transformants, pG14, pG17, and pG18, with single, two or multiple copies of interference cassettes inserted. As shown in Fig 2A, in all tested transformants *gna-1* mRNA was detected at significantly lower levels than in the wild-type strain. It should be noted that the lowest level of *gna-1* mRNA (only 7.7% of the mRNA of the wild-type) was detected in pG14 with a single insertion of the interference cassette. This qRT-PCR result clearly suggests that RNAi against the mRNA of *gna-1* happened in these transformants, although it is difficult to find a co-relationship between the copy number of the RNAi cassette and the interference outcome (Fig 2A).

Expression of *gna-1* and *pkaR* in double-silenced transformants (pGP) was also assessed with qRT-PCR. Both *gna-1* and *pkaR* transcripts were significantly decreased in transformants pGP1, pGP6 and pGP7 (Fig 2B). The mRNA level of *gna-1* in pGP1, pGP6 and pGP7 dropped to a proportion of 16.0%, 10.3% and 12.7%, respectively, of that in the wild-type strain (Fig 2A). Expression of *pkaR* was also examined and a similar decrease was observed in the mutants. The mRNA level of *pkaR* in pGP1, pGP6 and pGP7 dropped to a proportion of 29.8%, 12.7% and 22.0%, respectively, of that in the wild-type strain (Fig 2B). Thus, *gna-1* and *pkaR* were simultaneously knocked down in pGP1, pGP6 and pGP7.

### Knockdown of *gna-1* has significant effects on perithecium formation, melanin and ChA biosynthesis

**Fig 3:**
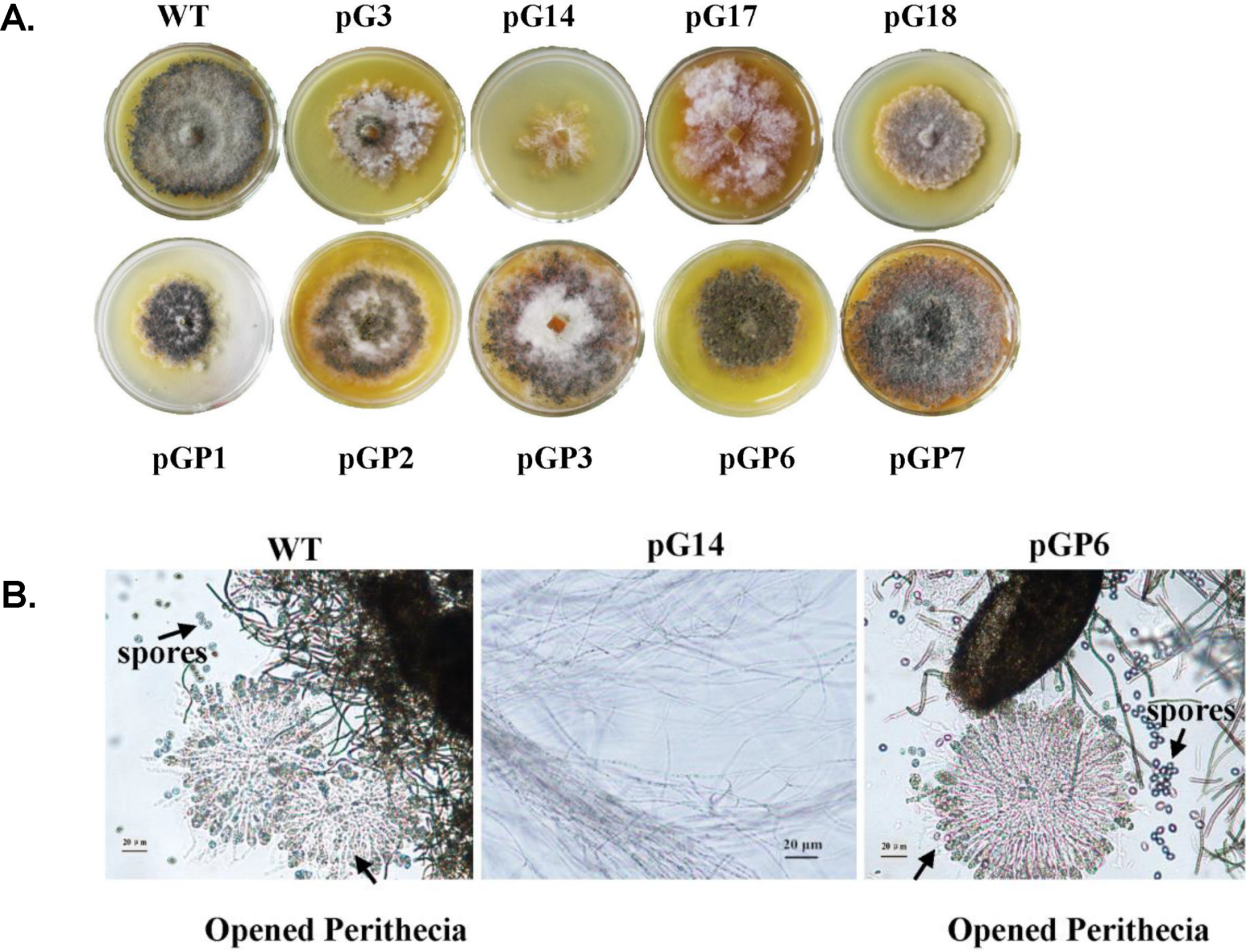
Knock-down of *gna-1* leads to deficiency in melanin, perithecia, and ChA production in *C*. *globosum*. **(A)** Colony morphology of silenced mutants of *gna-1*, or *gna-1/pkaR* double knock-down. All mutants were inoculated in PDA medium supplemented with 100 mg/L hygromycin B and incubated at 28°C for 9 days. **(B**) Light microscopy involving mycelium and perithecia formation in *C*. *globosum* NK102 (WT) and RNAi mutant pG14 and pGP6. Scale bar represents 20 μm.

**Fig 4:**
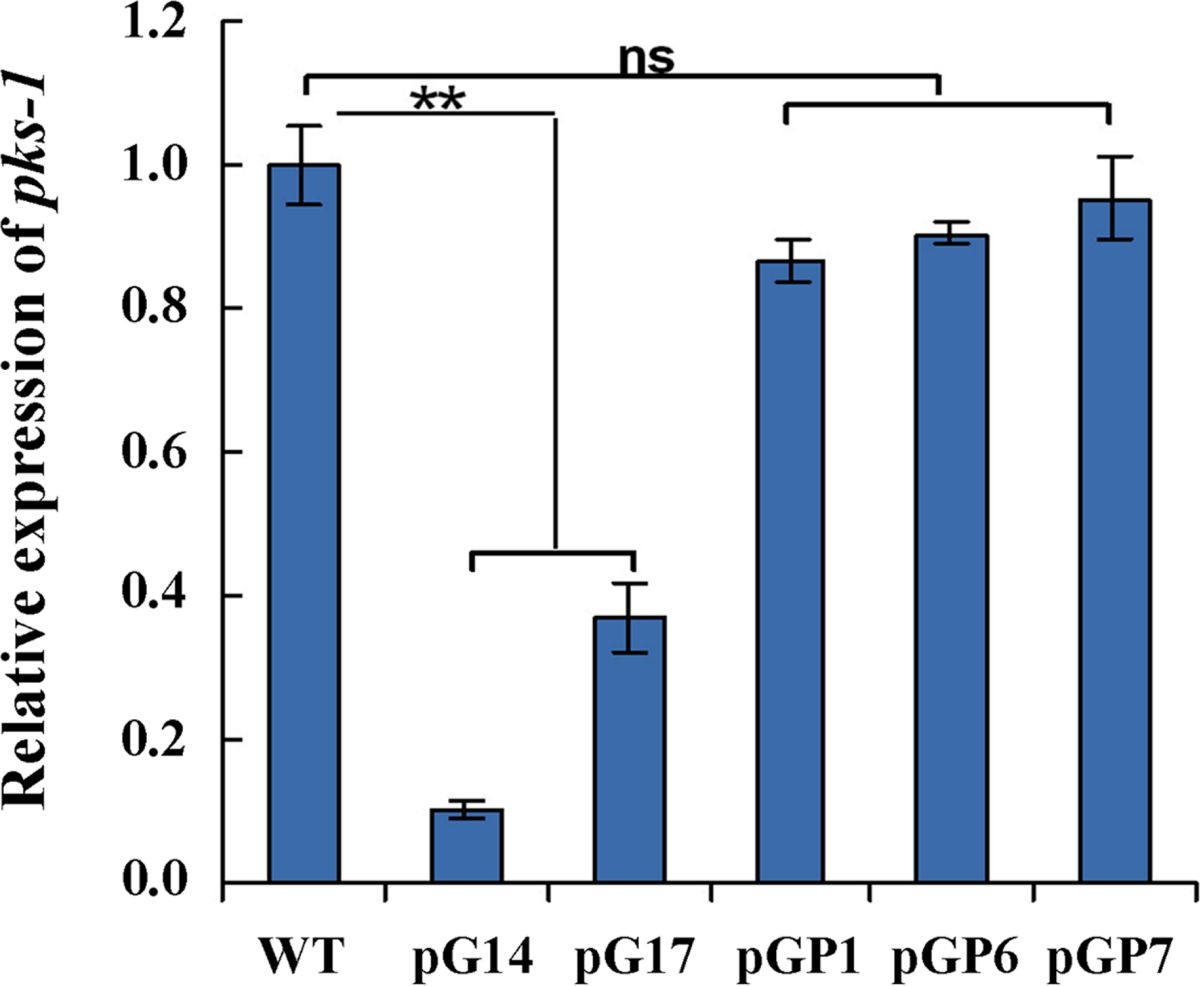
Gene expression of *pks-1* in RNAi mutants. Transcript levels of the *pks-1* were detected by qRT-PCR in the wild-type, pG and pGP transformants using the primers qPKS(s) and qPKS(as). There is significantly difference between the mutant and wild-type as indicated by two asterisks (p-value <0.01 with T-test analysis), ns: no significant difference. Experiments were performed in triplicate.

We found that the silenced transformants of *gna-1* displayed a distinct colony morphology from that of the wild-type (Fig 3A, upper panels). All of the pG transformants formed comparatively light pigmented colonies, an indication of less pigmentation of the mycelia and perithecia (fruiting body), particularly in pG14, which had the lowest level of *gna-1* mRNA (Fig 3A). Microscopic examination revealed that formation of perithecia in pG14 was almost abolished (Fig 3B). The transcript level of polyketide synthase gene, *pks-1*, which was previously proven to be essential for the biosynthesis of 1, 8-dihydroxynaphthalene (DHN) melanin in *C*. *globosum* NK102 [15], dramatically decreased in the mutant strains pG14 and pG17, to only approximately 37% in pG17 and 10.2% in pG14 (Fig 4). Hence, *gna-1* is critical for the development of fruiting bodies and melanin biosynthesis in *C*. *globosum* NK102.

**Fig 5:**
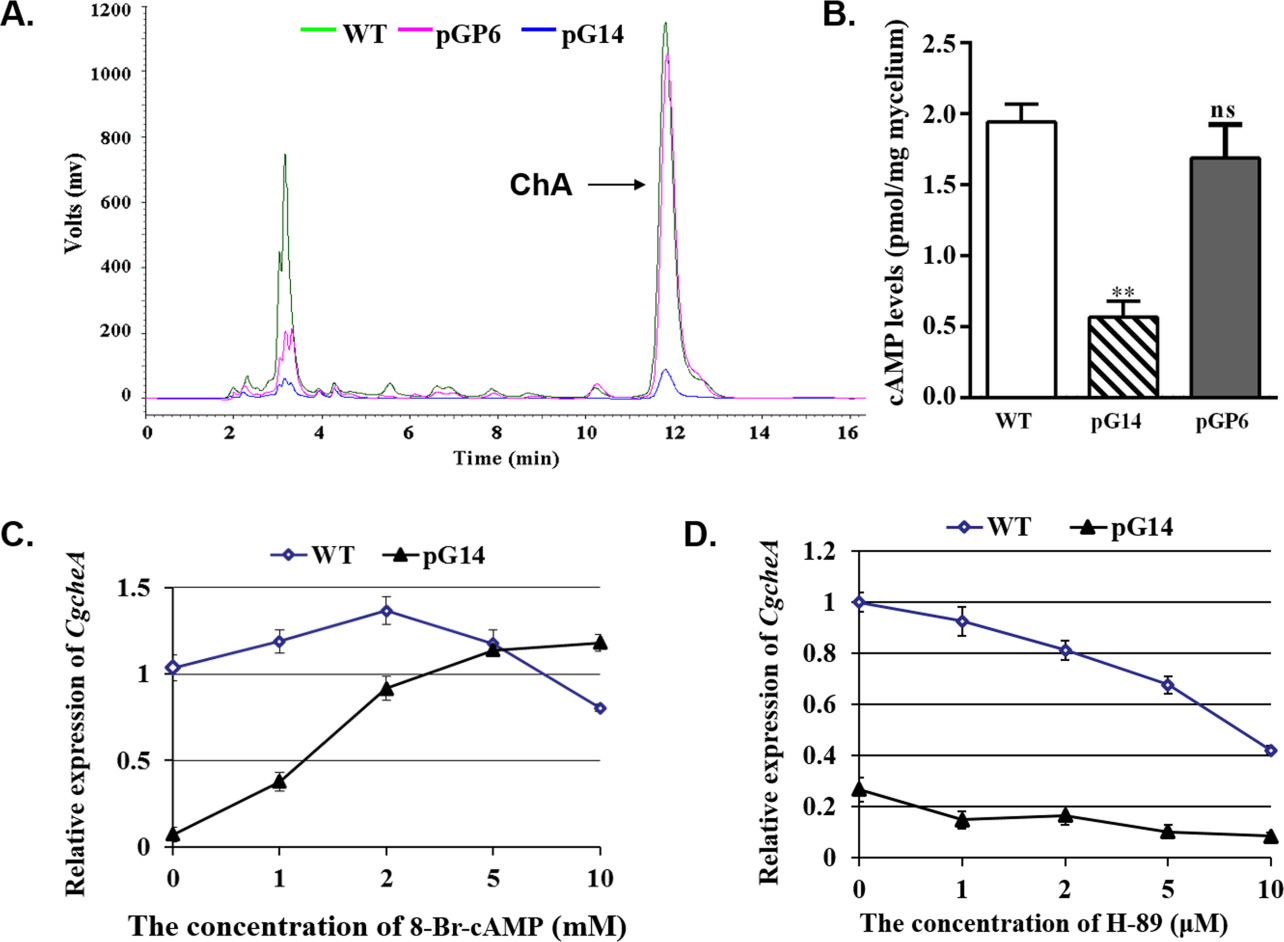
Diminished biosynthesis of ChA. **(A)** HPLC analysis on the production of ChA in the wild-type and the RNAi mutants. Arrow indicates the peak of ChA. **(B)** The cAMP assay for the silenced mutants. Relative expression of gene *CgcheA* from the wild-type (WT) and the transformant pG14 in the presence of 8-Br-cAMP**(C)** and H-89**(D)** at indicated concentrations.

To investigate whether the G-protein signaling pathway was involved in the production of ChA, a HPLC analysis was performed to quantify the concentration of ChA in each strain (S3 Appendix). In 8-day-old fermentation broths, yields of ChA were significantly lower in the *gna-1* silenced mutants compared to the wild-type strain (Fig 5A and Table 1). For instance, in pG14, the yield of ChA was only 4.17±1.70 mg/L, compared to 53.71± 4.34 mg/L in the wild-type (Table 1). This demonstrated that *gna-1* is critical for the biosynthesis of ChA.

**Table 1:** Yields of chaetoglobosin A in different strains.

Strain	Retention time (min)	Biomass (mycelia dry weight) (g/200ml)	ChA content (mg/L)
WT	11.812	2.25±0.31	53.71±4.34
WT+8-Br-cAMP	11.806	2.02±0.26	69.45±8.07
pG14	11.828	1.75±0.24	4.17±1.70
pG17	11.793	1.86±0.21	7.56±2.32
pGP1	11.812	2.38±0.32	56.80±10.04
pGP6	11.855	2.29±0.44	57.37±6.75
pGP7	11.841	2.06±0.23	55.68±7.36

8-Br-cAMP, 2 mM used. The data was mean ± standard deviation of three repeats.

### Defects in perithecia, melanin and ChA of pG mutants can be reversed by simultaneous knock-down of *gna-1* and *pkaR*

To investigate whether ChA biosynthesis was controlled by the downstream effector kinase PKA in the pathway, mutants of simultaneous knock-down of both *pkaR* (the regulatory subunit of the PKA kinase complex) and *gna-1* were made by the same RNAi strategy, to obtain the desired double knock-down transformants, *e*.*g*. pGP6 and pGP7 (Figure B in S2 Appendix). The qRT-PCR analysis confirmed a significant drop of both *gna-1* and *pkaR* mRNA in pGP1, pGP6 and pGP7 (Fig 2). Interestingly, these mutants showed recovered phenotype to some extent if compared to the initial deficient phenotype of the *gna-1* mutants. For instance, the selected pGP transformants produced melanin on the plates to form dark colonies (Fig 3A), and the formation of perithecia was also observed under the microscope (Fig 3B, right panel). Secondly, the diminished level of *pks-1* mRNA was restored to approximately wild-type level in pGP transformants, with 86.8% and 90.5%, 95.1% (versus the wild-type) for pGP1, pGP6 and pGP7, respectively (Fig 4). Biosynthesis of ChA in pGP transformants such as pGP1, pGP6 and pGP14, was also restored to the yield of the wild-type strain (Fig 5A, S3 Appendix and Table 1). The phenotype showed by the double knock-down mutants suggests that *pkaR* is the downstream effector of Gna-1 towards the biosynthesis of the melanin pigment and ChA. Namely, Gna-1 inhibits PkaR function, which negatively regulates sexual development, melanin production and ChA biosynthesis.

### The second messenger cAMP has an effect on *CgcheA* transcription and is positively regulated by Gα protein and negatively regulated by PKAR

One of the targets of the heterotrimeric G protein is the enzyme adenylyl cyclase that converts ATP to cAMP as a second messenger in filamentous fungi as in other eukaryotic organisms. We wondered whether diminished expression of the Gα subunit in *gna-1* mutants could affect synthesis of cAMP *in vivo*, which led to the defective phenotype of the mutant. To test this theory, we used EIA to determine cAMP concentrations in *gna-1* silenced mutant pG14 and wild-type strain mycelium extracts. As shown in Fig 5B, the extracts from pG14 exhibited a mean cAMP level of 0.57±0.11 pmol/mg mycelium, disclosing a striking 3.4-fold reduction (P<0.001) compared to the wild-type (1.94±0.13 pmol/mg). Furthermore, when 2 mM 8-Br-cAMP, an analog of cAMP that has a similar function to cAMP, was supplemented in the culture, the production of ChA from pG14 was clearly stimulated (Table 1). We further determined whether this was due to activation of expression of the gene *CgcheA* in the mutant pG14, with the wild-type *C*. *globosum* as a control. In the quantification by qRT-PCR, a pair of primers qCHGG_01239(s)/qCHGG_01239(as) were used (S1 Appendix). We found that 8-Br-cAMP restored expression of *CgcheA* in pG14, and could reach the wild-type level when the concentration of the chemical was above approximately 4 mM in the medium (Fig 5C). The *CgcheA* mRNA level continued to increase with the accretion of 8-Br-cAMP concentration to 10 mM (the highest amount tested in this study). Interestingly, a dual effect of 8-Br-cAMP was observed on the expression of *CgcheA* in the wild-type strain; when the concentration was below ~2 mM, 8-Br-cAMP stimulated transcription of *CgcheA*, but when it was above 2 mM, expression of *CgcheA* decreased (Fig 5C). These findings, therefore, demonstrated that the cAMP is positively regulated by Gα protein and has an important role on *CgcheA* transcription.

To determine whether the recovered phenotype appeared in *gna-1* and *pkaR* double silenced mutant was caused by a changed level of intracellular cAMP, the concentration of cAMP in pGP6 was measured as mentioned above. As expected, the mean cAMP level of pGP6 is 1.69±0.23 pmol/mg mycelium, although still below the normal level, disclosing a striking 2.98-fold increase compared to pG14 (Fig 5B). A PKA activity inhibitor H-89 [30] was also employed to assess the effects of PKA on ChA biosynthesis. As shown in Fig 5D, the mRNA level of *CgcheA* gene in pG14 was about one fourth of the wild-type stain. As the concentration of H-89 supplemented increasing, the gene expression reducing both in the wild-type and in the pG14. However, the decrease rate was much lower in pG14. When 10 μM (the highest amount tested in this study) H-89 was supplemented in the culture, the gap between the wild-type and pG14 reached the smallest. Thus, it is demonstrated that PKA negatively regulate cAMP level and has an important role on *CgcheA* transcription.

### Genome-wide profiling of gene expression in *gna-1* knock-down mutant pG14 by RNA-seq profiling

To assess a global profile of genes regulated by *gna-1*, an RNA-Seq profiling analysis was performed to identify the differentially expressed genes associated with the biosynthesis of ChA in the *gna-1* mutant pG14. Total RNA was extracted from mycelium grown in MCC media for 9 days for the Illumina HiSeq™ sequencing as described in Materials and Methods. The resulting sequences were aligned to the reference genome of *C*. *globosum* NK102 (unpublished data) and the information was used to analyze the DEGs between the wild-type strain and pG14. DEGs were selected based on the FDR <0.001 and |log_2_Ratio| ≥1. In a total of 3326 differentially expressed genes, 688 genes were up-regulated, while 2638 genes were down-regulated in pG14 (data in S4 Appendix).

**Fig 6:**
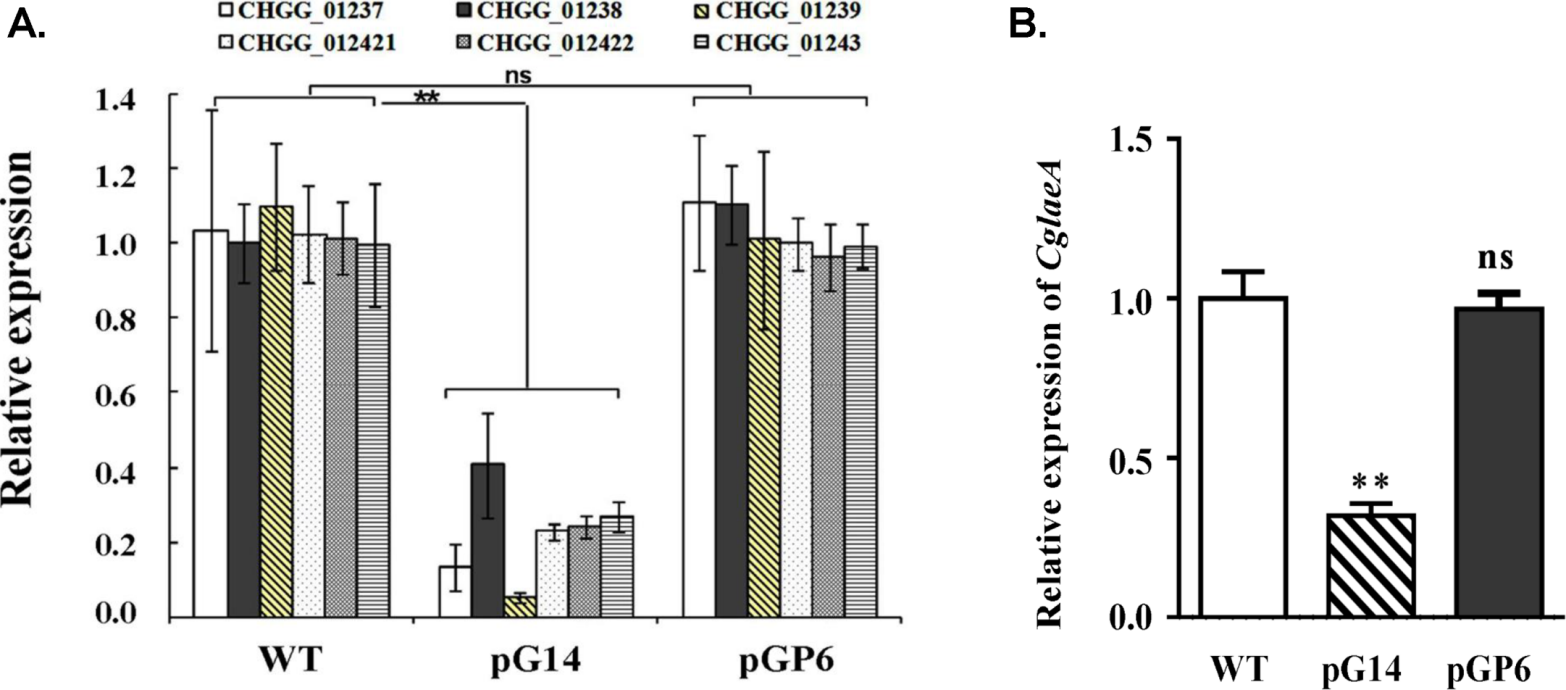
Quantification of the gene candidates likely involved in ChA biosynthesis by qRT-PCR. **(A)** Relative expression levels by qRT-PCR analysis of six key genes, chosen from the RNA-seq profiling and found in the chaetoglobosin biosynthetic gene cluster, in the transformants compared with the wild-type (WT) strain. The six genes are C6 zinc finger protein (CHGG_01237), transposase (CHGG_01238), PKS−NRPS hybrid gene cluster, *CgcheA* (CHGG_01239), P450 oxygenase (CHGG_012421), FAD-dependent oxidoreductase (CHGG_012422) and P450 oxygenase (CHGG_01243). All transcripts were normalized against *actin* cDNA amplified with primers qActin(s) and qActin(as) (S1 Appendix). **(B)** Decreased expression levels of *CglaeA* in pG14 and restoration in pGP6. There is significantly difference between the mutant and wild-type as indicated by two asterisks (p-value <0.001 with T-test analysis), ns: no significant difference. Experiments were performed in triplicate.

At least eight genes that were located in the gene cluster of *CgcheA* were down-regulated in pG14 (Table 2). Most of these genes were previously reported to be involved in ChA biosynthesis, including CHGG_01239 (*CgcheA*), CHGG_00542 (*pks-1*) and CHGG_01690 (*CglaeA*). The gene with the highest coefficient of variation (log_2_ ratio = -4.1) was CHGG_01239 (*CgcheA*), encoding an iterative PKS-NRPS presumably for synthesis of the skeleton structure of ChA, and displaying only 6% expression in pG14 compared to the wild-type. We further employed a qRT-PCR verification on the RNA-Seq result for seven characterized genes and the data was highly consistent with the DEG data (Fig 6A and Table 2). Transcription levels of all seven genes dropped dramatically in pG14 (approximately 3- to 20-fold), yet recovered to nearly the wild-type level in pGP6, in particular, LaeA, which is a global transcription regulator (Fig 6B). These RNA-seq and qRT-PCR results clearly demonstrate that the critical role of Gα in the biosynthesis of ChA. Furthermore the results indicate an interaction between the G protein pathway and the LaeA/VeA/VeB complex, and the histone acetyltransferase CgSptJ.

**Table 2:** Expression variation of genes putatively related to ChA biosynthesis detected by RNA-seq profiling.

Gene symbol	Deduced function (homolog)	Log_2_ ratio (pG14/WT)	FDR	Up- or down-regulation (pG14/WT)
CHGG_01237	C6 zinc finger protein	-1.9	5.26E-12	Down
CHGG_01239	*CgCheA*, PKS−NRPS hybrid	-4.1	0	Down
CHGG_01240	*CgCheB*, enoyl reductase	-2.5	7.19E-33	Down
CHGG_01241	hypothetical protein	-2.4	3.22E-41	Down
CHGG_01242–1	P450	-2.9	2.39E-62	Down
CHGG_01242–2	FAD-dependent oxidoreductase	-2.9	2.39E-62	Down
CHGG_01243	P450 (*gliF*)	-2.5	1.26E-44	Down
CHGG_01244	hypothetical protein	-2.5	8.75E-18	Down
CHGG_00542	*Pks-1*(*alb1*)	-2.8	0.066018	Down
CHGG_01690	*CgLaeA*	-1.7	0.000133	Down
CHGG_10370	*CgVeA*	-0.5	2.25E-13	Down
CHGG_09972	*CgSptJ*, histone acetyltransferase	-1.1	0.00173	Down

## Discussion

In the pharmaceutical arena, ChA has drawn attention as an anticancer and antimicrobial agent. To understand the control of the biosynthesis of ChA, we tried to block, via an established RNAi approach, the G protein-cAMP/PKA signal transduction pathway in *C*. *globosum* NK102, a high producer of ChA (~ 300 mg/kg in solid culture, unpublished data). Knock-down of the expression of *gna-1* which encodes an alpha-subunit homolog of the heterotrimeric G protein resulted in dramatic phenotypic changes in this fungus. The mycelia pigmentation of the mutant strains was almost halted, and as a result the colonies appeared light colored (Fig 3A, upper row of plates). Molecular evidence by qRT-PCR on the mRNA of *pks-1*, which was responsible for melanin biosynthesis [15], confirmed that expression of *pks-1* dropped sharply in the two tested mutants pG14 and pG17 (Fig 4). Secondly, we demonstrated that Gα function was critical for sexual stage development in this fungus as the formation of perithecia, the sexual fruiting body, was severely impaired in the knock-down mutant strain pG14 (Fig 3B). This observation is in a concomitance with previous findings that the G protein-mediated signal pathway is critical for sexual development in other filamentous fungi [17–21, 31]. More significantly, we found that biosynthesis of ChA in pG14 substantially decreased to a level approximately of one-tenth of that produced by the wild-type *C*. *globosum* NK102 (Fig 5A and Table 1). Consistent with this observation, expression of genes involved in ChA biosynthesis, *e*.*g*., the hybrid PKS−NRPS-encoding gene *CgcheA* (CHGG_01239), were dramatically down regulated in pG14 and pG17 by qRT-PCR or RNA-seq analysis (Fig 6A and Table 2). These data ascertain a positive role of Gα and the pathway in the sexual development and ChA biosynthesis in *C*. *globosum*. Furthermore, we demonstrated that the downstream effector of G protein was the cAMP/PKA signaling pathway regulating the biosynthesis of melanin and ChA, and sexual development. This was achieved by generating double knock-down mutants for both genes *gna-1* and *pkaR*, the latter encodes the regulatory subunit of cAMP-dependent PKA, *i*.*e*., we silenced simultaneously both genes *gna-1* and *pkaR* to obtain a set of mutant strains named pGPs. Double knock-down of the genes restored the defective phenotype of the *gna-1* mutants to about the wild-type phenotype (Figs 3A and 5A and S3 Appendix). For instance, pGP1-7 produced melanin (Fig 3A, bottom row of plates) and expression of *pks-1* in three tested strains (pGP1, pGP6 and pGP7) was restored to the level of the wild-type strain (Fig 4). Biosynthesis of ChA (Fig 5A, S3 Appendix, and Table 1) and the expression of related genes in the same gene cluster as *CgcheA* (Fig 6) were also restored to the wild-type level. These data suggest that Gna1 inhibits PkaR, which itself had a negative effect on the phenotypes observed in this study, including sexual development, and melanin and ChA biosynthesis. We then showed that knock-down of *gna-1* caused a deficiency in *in vivo* cAMP formation. The intracellular cAMP level in pG14 is significantly decreased compared to the wild-type strain (Fig 5B). When the cAMP analog, 8-Br-cAMP, was added to the media, expression of the key gene *CgcheA* that was responsible for ChA biosynthesis, was recovered in the mutant pG14 (Fig 5C). When the concentration of 8-Br-cAMP was over 2 mM, expression of *CgcheA* almost returned to the wild-type level. Interestingly, in the wild-type strain, *CgcheA* expression as well as ChA production were moderately affected by the supplement of 8-Br-cAMP (Fig 5C); when the concentration was over 2 mM, the chemical had an inhibitory effect on *CgcheA* expression. When *gna-1* and *pkaR* simultaneously silence, the intracellular concentration of cAMP was increased to 1.69±0.23 pmol/mg mycelium, approaching the normal level (1.94±0.13 pmol/mg). It is demonstrated that PKA is a downstream effector of Gna1. Further inhibitor supplementing experiment confirmed that the *CgcheA* expression was regulated by activity of PKA (Fig 5D). Thus, the heterotrimeric G protein/cAMP/PKA signaling pathway regulates ChA biosynthesis, pigmentation and sexual development in *C*. *globosum*.

The heterotrimeric G protein-activated cAMP/PKA signaling transduction pathways with regard to secondary metabolism regulation have been well studied in a number of model fungi. However, the pathway may function differently in different fungi. *Aspergillus nidulans* FadA (the α-subunit of the G protein) negatively regulates both asexual reproduction and sterigmatocystin/aflatoxin biosynthesis. Deletion of *fadA* resulted in activation of sterigmatocystin/aflatoxin biosynthesis [31, 32]. A repression effect of G protein α-subunit on secondary metabolism has also been reported in *Fusarium graminearum* [33]. Deletion of Ga resulted in increased deoxynivalenol and zearalenone production in this fungus. A similar example is observed with *F*. *fujikuroi* Ffg1 and Ffg3 (both stimulators of the cAMP cyclase), which also show a negative regulation on fusarubin biosynthesis [19]. On the other hand, positive effects of the pathway on secondary metabolism are found in some other fungi. For instance, constitutive activation of PGA1 in *Penicillium chrysogenum* caused an increase in the production of penicillin, chrysogenin and roquefortine [34]. One of our previous works demonstrated that the counterpart of Gα in the taxol-producing fungus *Pestalotiopsis microspore* resulted in a sharp decrease in pestalotiollide B production while introduction of extra copies of *pgα1* led to enhanced production of pestalotiollide B [21]. In this paper, we demonstrated that group I Gα protein encoded by *gna-1* has a positive effect on melanin and ChA biosynthesis in *C*. *globosum*.

**Fig 7:**
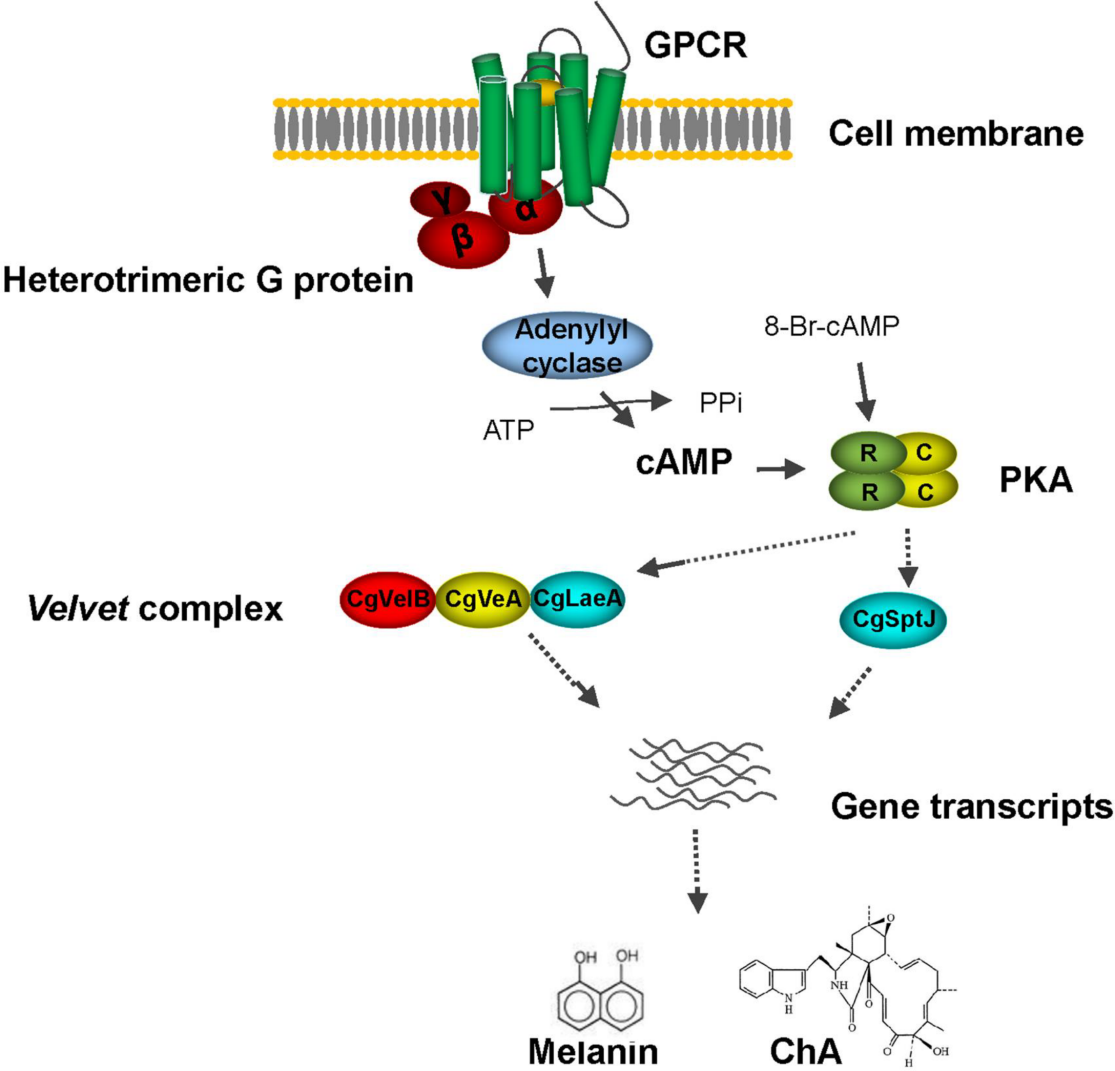
Suggested schematic on the regulatory network responsible for ChA and melanin biosynthesis of *C*. *globosum*.

In filamentous fungi, the regulatory networks of secondary metabolism are complex. Other pathways are also critically involved. For example, a conserved global regulatory unit, the *velvet* complex, composed of mainly LaeA, VeA and VelB, is reported to be involved in development and secondary metabolism in numerous fungi, including *Aspergillus* spp., *Trichoderma reesei*, *Penicillium chrysogenum* and *F*. *verticillioides* [35–39]. The roles of VeA and LaeA on secondary metabolism in *C*. *globosum* was confirmed by studies of Watanabe and Zou teams [24, 40]. Deletion of either *CglaeA* or *CgVeA* led to a significant absence of many secondary metabolites including ChA [24], whereas overexpression of *CglaeA* upregulated expression of the chaetoglobosin gene cluster and resulted in the isolation of a new cytochalasin, chaetoglobosin Z [40], demonstrating positive regulatory activity of *CglaeA* and *CgVeA* on ChA biosynthesis in *C*. *globosum*. It is worth noting that in this study, expression of *CglaeA* was down regulated (approximately 68.1%) in *gna-1* silent mutants and restored to wild-type level when *pkaR* was silenced simultaneously (Fig 6B), suggesting that *CglaeA* is somehow affected by heterotrimeric G protein activated cAMP/PKA signaling pathway. Consistent with qRT-PCR data, transcription levels of *CglaeA* and *CgVeA* are all reduced in pG14 (log_2_ ratio = -1.7 for *CglaeA*, log_2_ ratio = -0.5 for *CgVeA*) compared to the wild-type strain (Table 2). In addition, a previous study suggests that biosynthesis of ChA was also affected by a histone acetyltransferase CgSptJ; ChA production was sharply down regulated to undetectable levels when *CgSptJ* was deleted [24]. In this work, expression of *CgSptJ* was significantly down regulated in the Gα protein silenced mutant pG14 as verified by both RNA-seq analysis (log_2_ ratio = -1.1) (Table 2) and qRT-PCR (Fig 6A). With our findings, we hypothesize that CglaeA, CgVeA and CgSptJ probably work as downstream effectors that dictate expression of the ChA biosynthesis gene cluster by interacting with G protein/cAMP/PKA signaling (Fig 7).

In summary, our study, for the first time, provides information on the effects of the G protein-cAMP/PKA pathway on ChA biosynthesis and sexual development in *C*. *globosum*. As there are approximately 40 gene clusters that are likely involved in the biosynthesis of secondary metabolites in the genome of *C*. *globosum* CBS148.51 [39], unraveling signal transduction in this fungus will help with understanding the regulatory networks, further with facilitating the metabolic engineering to improve the yield of ChA and with hunting other novel secondary metabolites.

## Supporting information

S1 Appendix Primers used in the study. (DOCX)

S2 Appendix Southern blotting analysis for integration of the interference vectors pGNA (A and C) and pGNA-PKAR (B). **(A)** Genomic DNA extracted from pG transformants was digested with *Xba* I and approximately 5 μg digested DNA was loaded onto 0.8% agarose. Fragments of the correct sizes (8.0 kb and 2.7 kb) were detected with the labeled *gna-1* fragment (shown as a green bar) in RNAi cassette. WT, wild-type strain. **(B)** Genomic DNA extracted from pGP transformants was digested with *Xba* I and detected with the labeled *gna-1* fragment (shown as a green bar) in RNAi cassette. Predicted correct bands at 8.0 kb and 3.3 kb are indicated. WT, wild-type strain. **(C)** Genomic DNA extracted from pG transformants was digested with *Xho* I and detected with linearized pSilent-1. WT, wild-type strain.
(DOCX)

S3 Appendix HPLC analysis on the production of ChA in the wild-type and the RNAi mutant pG14, pGP1, pGP6 and pGP7. The retention time of ChA is 11.8 min.
(DOCX)

S4 Appendix Summary of all genes that have a difference expression between WT and pG14. Data is obtained by RNA-seq profiling analyses. "CG1" indicates the wild-type of *Chaetomium globosum* NK102. "CG2" indicates *gna-1* knock-down mutant pG14. The table include 14 columns, meanings of each column are as follow: Gene length: Length of all exon in gene; CG1-Expression: UniqReads of aligned reads in sample CG1; CG2-Expression: UniqReads of aligned reads in sample CG2; CG1-RPKM: RPKM value of sample CG1; CG2-RPKM: RPKM value of sample CG2; log_2_ Ratio(CG2/CG1): log_2_(CG2-RPKM/CG1-RPKM), log_2_(Fold Change); Up-Down-Regulation(CG2/CG1): CG2 is a Up/down Regulation relative to CG1; P-value: p value for hypothesis testing; FDR: False Discovery Rate.
(XLSX)
