# Supplementary material for: Gα-cAMP/PKA pathway positively regulates pigmentation, chaetoglobosin A biosynthesis and sexual development in Chaetomium globosum
Source: PLoS One. 2018 Apr 13;13(4):e0195553. doi: 10.1371/journal.pone.0195553 (PMC5898716; doi:10.1371/journal.pone.0195553)
Supplement: S1 Appendix — (DOCX) [file pone.0195553.s001.docx]

**S1 Appendix**

Gα-cAMP/PKA pathway positively regulates pigmentation, chaetoglobosin A biosynthesis and sexual development in *Chaetomium globosum*

Yang Hu^1^, Xiaoran Hao^2^*, Longfei Chen^3#a^, Oren Akhberdi^3^, Xi Yu^3#b^, Yanjie Liu^4^, Xudong Zhu^4^*

^1^ Department of Pathogen Biology, School of Basic Medical Sciences, Tianjin Medical University, Tianjin, China.

^2^ National Experimental Teaching Demonstrating Center, School of Life Sciences, Beijing Normal University, Beijing, China.

^3^ Department of Microbiology, College of Life Sciences, Nankai University, Tianjin, China.

^4^ Beijing Key Laboratory of Genetic Engineering Drug and Biotechnology, Institute of Biochemistry and Biotechnology, School of Life Sciences, Beijing Normal University, Beijing, China.

^#a^ Current Address: Department of Biopharmaceutical, Xinchang Pharmaceutical Factory, Zhejiang Medicine Co., LTD, Shaoxing, China.

^#b^ Current Address: Department of of Microbiology, Institute for Applied Biosciences, Karlsruhe Institute of Technology, Karlsruhe, Germany.

* Corresponding author

E-mail: zhu11187@bnu.edu.cn (ZX) or 2015xrhao@bnu.edu.cn (HX)

**Primers used in the study.**

| **Primer name** | **Sequence (5′-3′)** |
| --- | --- |
| GNA1(s) | ATTA CTCGAG GGTACC ATCGCTCGTATCGCTGCGC |
| GNA1(as) | GGCGC AAGCTT AGATCT ATCGAGTCGAAGAGGGTGAG |
| GNA-PKAR(as) | CGTTT AAGCT TGGGT AAACC GATCG AGTCG AAGAGGGTGAG |
| PKAR(s) | GCAAA TTCGA ACCCA TTTGG CTCTTCCAGGCTC |
| PKAR(as) | GTCCA AAGCTT AGATCT GCGGGATAGCCTTCTGACACTG |
| Hyg(s) | ATGAAAAAGCCTGAACTCAC |
| Hyg(as) | GCAAAGTGCCGATAAACAT |
| qActin(s) | AACCGAGGCTCCCATCAAC |
| qActin(as) | TCACGGACGATTTCACGCTC |
| qGNA1(s) | CGTATCACGGAGACGACCTTC |
| qGNA1(as) | GGAGTAGTTGTTCATCGGGCTG |
| qPKAR(s) | CAGGGGTTCGTTGCTATCCAG |
| qPKAR(as) | CAATCTTGGACCGCTCGTAGG |
| qPKS(s) | ATCTTTCCGCCTAACCCGA |
| qPKS(as) | GTCCTTCGTTTCTGGGTTGTC |
| qCHGG_01237(s) | CCTGCAAACGATGCCTCAAG |
| qCHGG_01237(as) | TATCACCGGTTTTGGCTGCT |
| qCHGG_01238(s) | TACCAGCCAACTCGCGTAAG |
| qCHGG_01238(as) | GCAGGTGGTTGGCCATATCT |
| qCHGG_01239(s) | CCAGGAGCACGATACCATCTACAC |
| qCHGG_01239(as) | GGCATCCCCATGTAGTTCGTATTG |
| qCHGG_012421(s) | GGCTTCCAAAGCATACGCAG |
| qCHGG_012421(as) | TGGTGTTTGTTCGGGTCTCC |
| qCHGG_012422(s) | GCCGTGTCCGAGAGTTATGT |
| qCHGG_012422(as) | AGAGGAAAGCCAGCAGTTCG |
| qCHGG_01243(s) | AGGAAAGCTGGCATTGACGA |
| qCHGG_01243(as) | AGGTAGTCGCGAATGAGCTG |
| qLaeA(s) | TCATAGTGCTCCCCTCCACA |
| qLaeA(as) | AGGTCAACGCCGACATGAAT |
