# Supplementary material for: Gα-cAMP/PKA pathway positively regulates pigmentation, chaetoglobosin A biosynthesis and sexual development in Chaetomium globosum
Source: PLoS One. 2018 Apr 13;13(4):e0195553. doi: 10.1371/journal.pone.0195553 (PMC5898716; doi:10.1371/journal.pone.0195553)
Supplement: S2 Appendix — (A) Genomic DNA extracted from pG transformants was digested with Xba I and approximately 5 μg digested DNA was loaded onto 0.8% agarose. Fragments of the correct sizes (8.0 kb and 2.7 kb) were detected with the labeled gna-1 fragment (shown as a green bar) in RNAi cassette. WT, wild-type strain. (B) Genomic DNA extracted from pGP transformants was digested with Xba I and detected with the labeled gna-1 fragment (shown as a green bar) in RNAi cassette. Predicted correct bands at 8.0 kb and 3.3 kb are indicated. WT, wild-type strain. (C) Genomic DNA extracted from pG transformants was digested with Xho I and detected with linearized pSilent-1. WT, wild-type strain. (DOCX) [file pone.0195553.s002.docx]

**S2 Appendix**

Gα-cAMP/PKA pathway positively regulates pigmentation, chaetoglobosin A biosynthesis and sexual development in *Chaetomium globosum*

Yang Hu^1^, Xiaoran Hao^2^*, Longfei Chen^3#a^, Oren Akhberdi^3^, Xi Yu^3#b^, Yanjie Liu^4^, Xudong Zhu^4^*

^1^ Department of Pathogen Biology, School of Basic Medical Sciences, Tianjin Medical University, Tianjin, China.

^2^ National Experimental Teaching Demonstrating Center, School of Life Sciences, Beijing Normal University, Beijing, China.

^3^ Department of Microbiology, College of Life Sciences, Nankai University, Tianjin, China.

^4^ Beijing Key Laboratory of Genetic Engineering Drug and Biotechnology, Institute of Biochemistry and Biotechnology, School of Life Sciences, Beijing Normal University, Beijing, China.

^#a^ Current Address: Department of Biopharmaceutical, Xinchang Pharmaceutical Factory, Zhejiang Medicine Co., LTD, Shaoxing, China.

^#b^ Current Address: Department of of Microbiology, Institute for Applied Biosciences, Karlsruhe Institute of Technology, Karlsruhe, Germany.

* Corresponding author

E-mail: zhu11187@bnu.edu.cn (ZX) or 2015xrhao@bnu.edu.cn (HX)

**A.
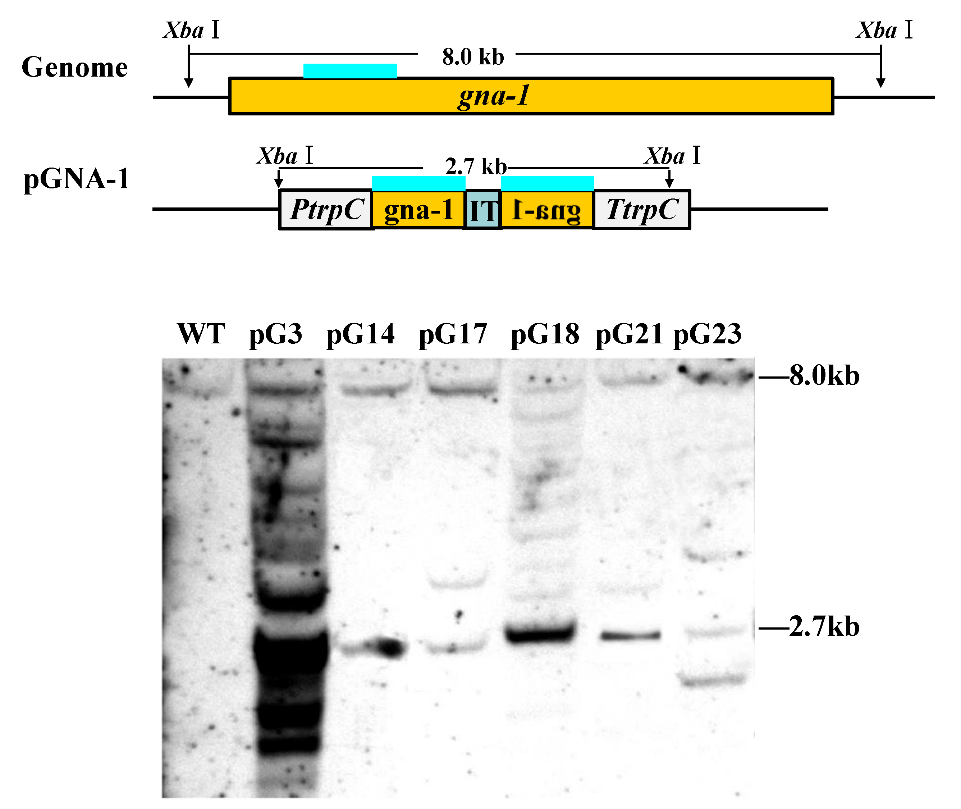
**

**B.
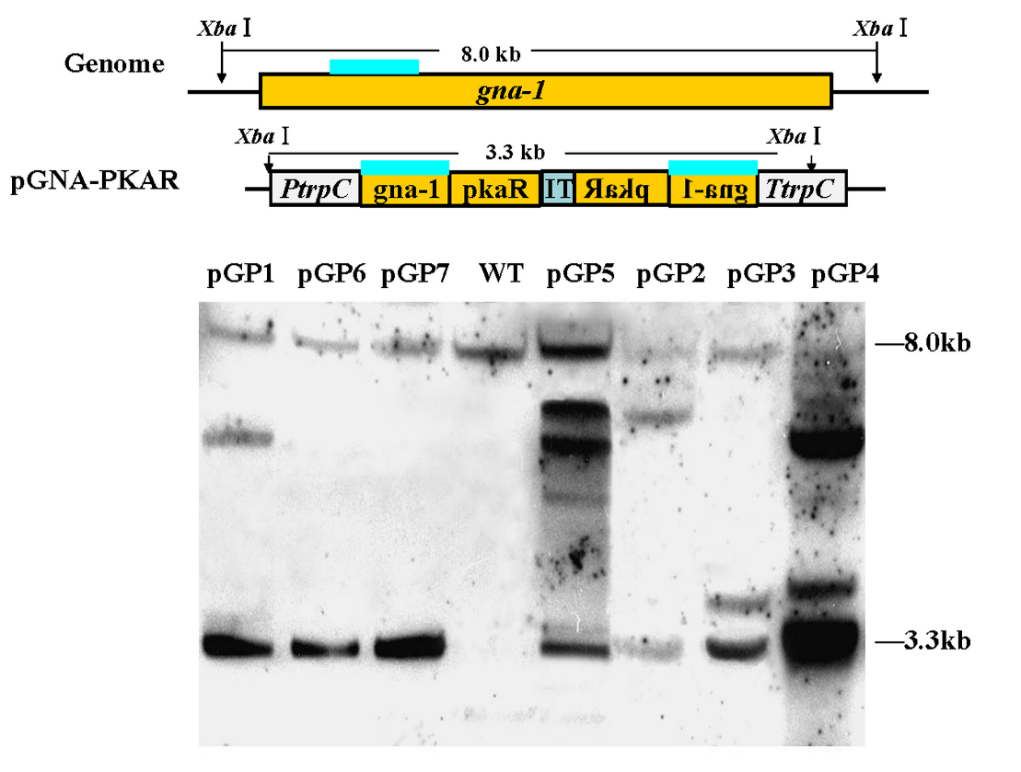
**

**C.
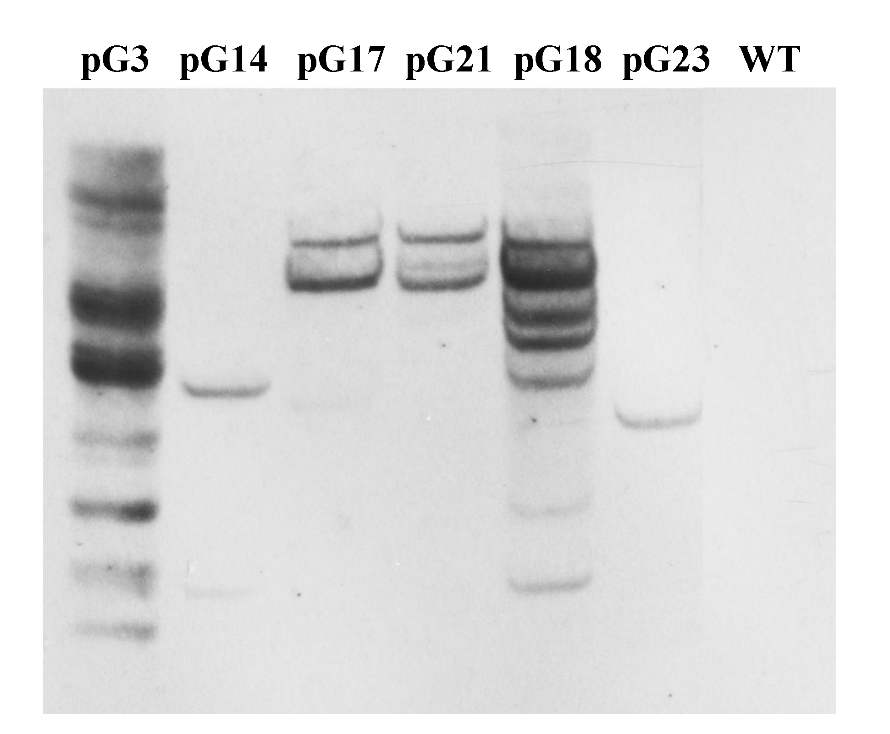
**
