# Supplementary material for: Gα-cAMP/PKA pathway positively regulates pigmentation, chaetoglobosin A biosynthesis and sexual development in Chaetomium globosum
Source: PLoS One. 2018 Apr 13;13(4):e0195553. doi: 10.1371/journal.pone.0195553 (PMC5898716; doi:10.1371/journal.pone.0195553)
Supplement: S3 Appendix — The retention time of ChA is 11.8 min. (DOCX) [file pone.0195553.s003.docx]

**S3 Appendix**

Gα-cAMP/PKA pathway positively regulates pigmentation, chaetoglobosin A biosynthesis and sexual development in *Chaetomium globosum*

Yang Hu^1^, Xiaoran Hao^2^*, Longfei Chen^3#a^, Oren Akhberdi^3^, Xi Yu^3#b^, Yanjie Liu^4^, Xudong Zhu^4^*

^1^ Department of Pathogen Biology, School of Basic Medical Sciences, Tianjin Medical University, Tianjin, China.

^2^ National Experimental Teaching Demonstrating Center, School of Life Sciences, Beijing Normal University, Beijing, China.

^3^ Department of Microbiology, College of Life Sciences, Nankai University, Tianjin, China.

^4^ Beijing Key Laboratory of Genetic Engineering Drug and Biotechnology, Institute of Biochemistry and Biotechnology, School of Life Sciences, Beijing Normal University, Beijing, China.

^#a^ Current Address: Department of Biopharmaceutical, Xinchang Pharmaceutical Factory, Zhejiang Medicine Co., LTD, Shaoxing, China.

^#b^ Current Address: Department of of Microbiology, Institute for Applied Biosciences, Karlsruhe Institute of Technology, Karlsruhe, Germany.

* Corresponding author

E-mail: zhu11187@bnu.edu.cn (ZX) or 2015xrhao@bnu.edu.cn (HX)

WT

pG14

pGP1

pGP6

pGP7
